# Systematic identification of therapeutic targets for coronary artery calcification: an integrated transcriptomic and proteomic Mendelian randomization

**DOI:** 10.3389/fcvm.2024.1419440

**Published:** 2024-10-25

**Authors:** Lihong Chen, Xiaoqi Ye, Yan Li, Xingwu Ran

**Affiliations:** ^1^ Department of Endocrinology & Metabolism, West China Hospital, Sichuan University, Chengdu, China; ^2^ Innovation Research Center for Diabetic Foot, Diabetic Foot Care Center, West China Hospital, Sichuan University, Chengdu, China

**Keywords:** coronary artery calcification, Mendelian randomization, target identification, genetic epidemiological study, vascular calcification

## Abstract

**Background:**

Coronary artery calcification (CAC) is associated with an increased risk of mortality and cardiovascular events. However, none therapeutic drugs have been proven effective for CAC treatment. The objective of this study was to identify potential therapeutic targets for CAC through the utilization of Mendelian randomization (MR) and colocalization analysis.

**Methods:**

The expression quantitative trait loci (eQTLs) of 16,943 genes from the eQTLGen consortium and protein quantitative trait loci (pQTLs) of 4,412 proteins from a plasma proteome were utilized as genetic instruments. Genetic associations with CAC were derived from a GWAS meta-analysis of 26,909 individuals. The MR and colocalization analysis were utilized to identify potential target genes.

**Results:**

A total of 671 genes were found to be significantly associated with the risk of CAC based on transcriptomic MR analysis at a false discovery rate <0.05, while proteomic MR analysis identified 15 genes with significant associations with CAC at the same threshold. With robust evidence from colocalization analysis, we observed positive associations between CWF19L2, JARID2, and MANBA and the risk of CAC, while KLB exhibited an inverse association. In summary, our study identified 23 potential therapeutic targets for CAC. Further downstream analysis revealed IGFBP3, ABCC6, ULK3, DOT1L, KLB and AMH as promising candidates for repurposing in the treatment of CAC.

**Conclusion:**

The integrated MR analysis of transcriptomic and proteomic data identified multiple potential drug targets for the treatment of CAC. ULK3, DOT1L, and AMH were recognized as novel targets for drug repurposing for CAC and deserve further investigation.

## Introduction

Cardiovascular disease is the predominant cause of morbidity and mortality on a global scale, affecting millions of individuals worldwide (1, 2). It is estimated that approximately 18.4 million deaths in 2019 were attributed to cardiovascular disease (1). The presence of coronary artery calcification (CAC), a prominent characteristic of atherosclerosis (3), holds significant predictive value for coronary artery stenosis with an impressive sensitivity rate of 97% and a specificity rate of 72.4% (4). CAC is strongly associated with future occurrences of coronary heart disease and mortality rates as well (5–7). Furthermore, CAC exhibits associations with various non-cardiovascular diseases including dementia, stroke, and cancer (8–10). However, currently, the availability of therapeutic drugs specifically targeting CAC remains limited.

The presence and extent of CAC can be evaluated using computed tomography. CAC can be classified into two main groups, namely medial arterial calcification and intimal calcification, based on the location and deposition site of calcium (11). These two groups have distinct etiologies and implications. Although the mechanism underlying CAC remains unclear, previous GWAS studies have identified several genetic loci associated with CAC, including CDKN2B-AS1, PHACTR1, APOB, ARSE and MMP16 (12–15). Understanding of genetic underpinnings of CAC may facilitate the development of novel therapeutic strategies. For example, extensive literature supports the pivotal role of ABCC6 in soft tissue calcification (16). Preclinical studies also reveal that KLB can be a target of vascular calcification (17).

Mendelian randomization (MR) employs genetic variation as exposures to address causal effect estimations. The allocation of genetic variants at conception minimizes the influence of environmental confounding factors, making MR estimates less susceptible to their effects (18, 19). A previous study conducted by Kavousi and colleagues utilized eQTL summary data from 600 coronary artery disease patients in the STRANET cohort as exposure variables to identify causal genes and explore the potential druggability of these gene targets. They successfully identified 11 genes located at independent risk loci (ENPP1/ENPP3, IGFBP3, MTAP, CDKN2A, CDKN2B, CXCL12, ARID5B, ADK and FGF23, COL4A1/2, and, APOE). Targeting drugs towards these genes may hold repurposing potential for CAC (14). MR can be utilized to inform drug repurposing.

Therefore, repurposing current available drugs can facilitate the development of therapies of CAC. Due to the publication of current largest multi-ancestry GWAS meta-analysis of CAC (14), it became feasible to investigate the potential therapeutic targets for CAC. In this study, we integrated transcriptomic and proteomic data as exposures to perform a MR and colocalization analysis for CAC, aiming to identify genes that were associated with this disease and to investigate the repurposing potential of drugs targeting these genes.

## Methods

### Study design

**Figure 1:**
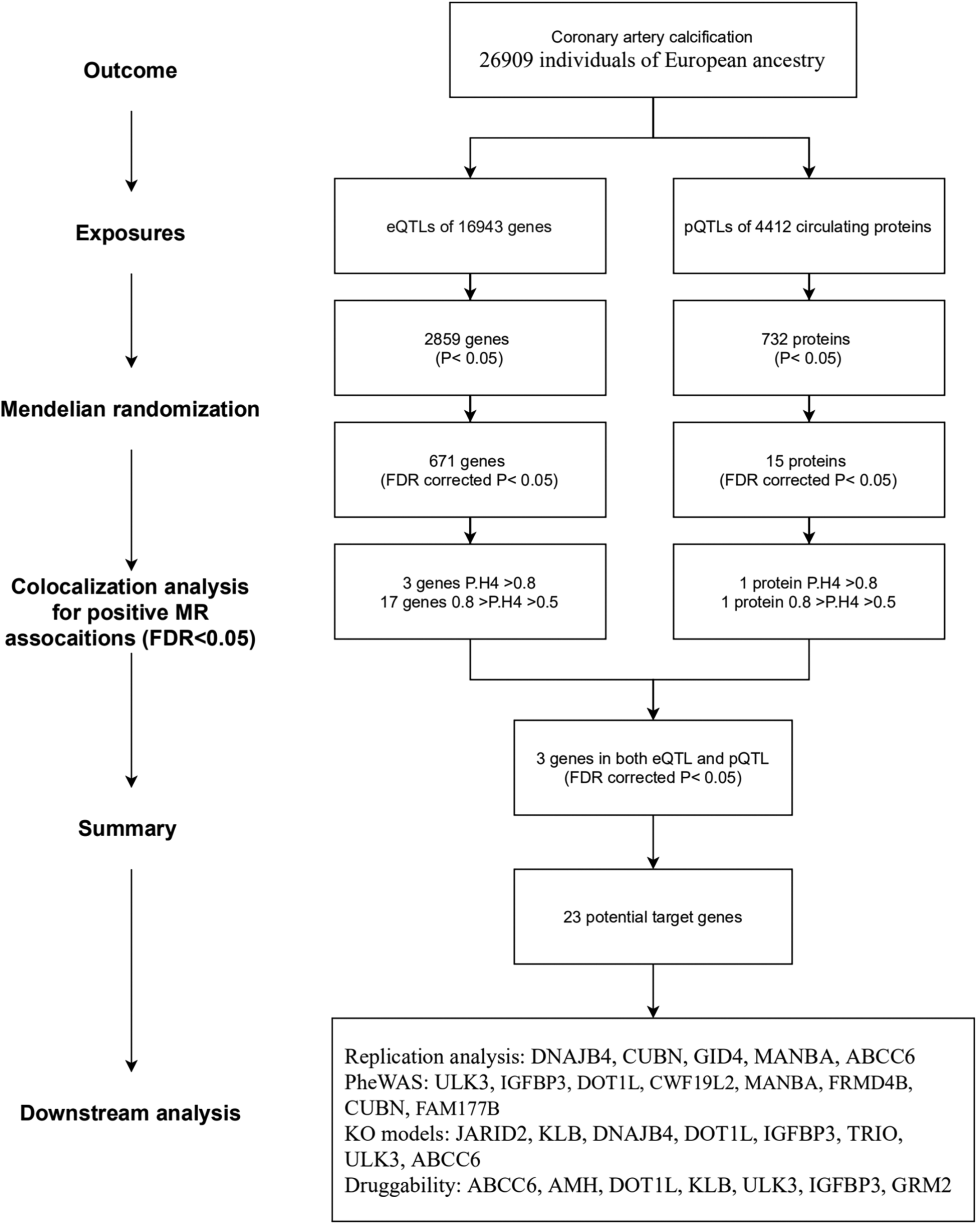
Overview of the study flow, analytic approaches, and key findings.

The overall study flow is illustrated in Figure 1. This study is based on publicly available summary-level genome-wide association studies (GWAS). The details of the GWAS studies are provided in Supplementary Table S1. Ethical approval was approved by relevant ethical review committees. The reporting of this study adheres to the Strengthening the Reporting of Observational Studies in Epidemiology guideline for MR studies (STROBE-MR statement) (20).

## Instrumental selection of exposure

### eQTL dataset

We utilized expression quantitative trait locus (eQTLs) as the proxy of exposure. Only cis-SNPs that exhibited an association with mRNA expression (*r*^2^ < 0.1, FDR corrected *P* value < 0.05) within a 1Mb proximity of each gene were selected. The summary-level data of eQTLs were obtained from the eQTLGen Consortium (21).

### pQTL dataset

Genetic associations for pQTLs with 4,907 circulating proteins were derived from what is, to the best of our knowledge, the largest GWAS conducted on plasma proteome in a cohort of 35,559 individuals from iceland (22). In brief, the measurement of these proteins was performed using the SomaScan version 4 assay. The rank-inverse normal transformed levels of proteins were adjusted with age and sex. Genome-wide association analysis was performed using the BOLT-LMM linear mixed model. For detailed information, please refer to the original article. We identified cis-pQTLs (*P* < 1.8*10^−9^, within 1Mb from the transcription start site of each gene) for subsequent MR analysis.

### Outcome data

Genetic associations for CAC were obtained from the summary results of a meta-analysis, which represents the largest multi-ancestry GWAS meta-analysis conducted on CAC to date (14). This comprehensive analysis included data from 16 cohorts, encompassing a total of 26,909 individuals of European ancestry and 8,867 individuals of African ancestry. The CAC quantity [log (CAC + 1)] within each cohort was modeled using linear regression with an additive genetic model, while adjusting for age, sex, and up to 10 principal components. A fixed-effects meta-analysis was conducted to combine the results of all GWAS. The summary statistics from each study were combined using an inverse variance-weighted (IVW) meta-analysis, with exclusion of variants exhibiting heterogeneity *I*^2^ ≥ 70% in the meta-analysis. We used the summary results of the European ancestry as the outcome.

We also replicated the MR analysis by obtaining genetic associations for abdominal aorta calcification (23). The GWAS summary statistics of abdominal aorta calcification in the UK biobank imaging cohort, comprising 31,785 individuals, were reported by Sethi and colleagues and accessed via the GWAS catalog (24).

### Mendelian randomization and colocalization analysis

In this study, both the gene expression and protein levels were employed as instrumental variables to capture potential therapeutic targets. Two-sample MR analysis was conducted to evaluate the associations between these instruments and CAC. The wald ratio or inverse-variance weighted (IVW) method was employed for single-variant or multi-variant MR analyses, respectively. Multiple testing was adjusted using the Benjamini–Hochberg method and a false discovery rate (FDR)- corrected *P*-value of <0.05 was considered statistically significant. MR results with a *P*-value < 0.05 but an FDR-corrected *P*-value > 0.05 were considered nominal significant.

To evaluate whether the genetic associations of exposure and outcome share the same causal variant, colocalization analysis was conducted for significant MR findings (25). We employed default priors with a probability of 1 × 10^−4^ for a single nucleotide polymorphism (SNP) being associated with trait 1 and trait 2, and a probability of 1 × 10^−5^ for an SNP being associated with both two traits simultaneously. This Bayesian approach evaluates the support for five hypotheses: (1) no association with either trait (H0); (2) association with only trait 1 (H1); (3) association with only trait 2 (H2); (4) association with both traits but involving different variants (H3); and finally, (5) association with both traits with same variant (H4). A posterior probabilities ≥ 0.8 for H4 (PP.H4) indicates strong evidence supporting colocalization, while a PP.H4 value between 0.5 and 0.8 was considered suggestive support of colocalization.

### Summary of findings

The findings from the MR analysis and colocalization were summarized. The results of the MR are generally more liberal (higher probability of false positive), while the results of colocalization analysis are more conservative. Therefore, MR was used to investigate potential therapeutic targets for CAC, while colocalization is used as a sensitivity analysis for MR. Potential therapeutic targets were identified based on a FDR-corrected *P* value of <0.05 in MR analysis. Three tiers were defined as follows: (1) Tier 1, targets with PP.H4 ≥ 0.8 in both eQTL and pQTL analysis; (2) Tier 2, targets with PP.H4 ≥ 0.8 in either eQTL or pQTL based analysis; and (3) Tier 3, targets with PP.H4 ≥ 0.5 in either eQTL or pQTL based analysis but lacking colocalization support (PP.H4 < 0.5), or exhibiting positive MR results in both eQTL and pQTL-based MR analysis.

### Phenome-wide association analysis

We searched PhenoScanner for phenotypes that were associated with the potential therapeutic targets, using a significance threshold of *P*-value < 1.0 × 10−8 (26).

### Gene knockout mice phenotype

We searched the phenotypes of gene knockout mice in two databases, namely the International Mouse Phenotyping Consortium (IMPC) (https://www.mousephenotype.org/) (27) and the Mouse Genome Informatics (MGI) (https://www.informatics.jax.org/). The phenotypes associated with the cardiovascular system or identified as risk factors were utilized to ascertain potential phenotypes of therapeutic target genes.

### Druggability of identified therapeutic targets

To evaluate the druggability of the identified targets, we acquired drug-gene interactions data from the Drug-Gene Interaction Databse (DGIdb v5.0.3) (28). The DGIdb (www.dgidb.org) is a web-based resource that offers curated information on drug-gene interactions and druggable genes, sourced from publications, databases, and other online platforms. In this study, we obtained the “interactions” file (released at 2023-Dec).

### Statistical software and methods

The statistical analyses were conducted using R version 4.3.1, and multiple testing was adjusted for FDR (false discovery rate) at a significance level of <0.05. The following packages were employed: “TwoSampleMR”, “coloc”, “forestplot”, “phenoscanner”, and “pheatmap”.

## Results

### eQTL based analysis

**Figure 2:**
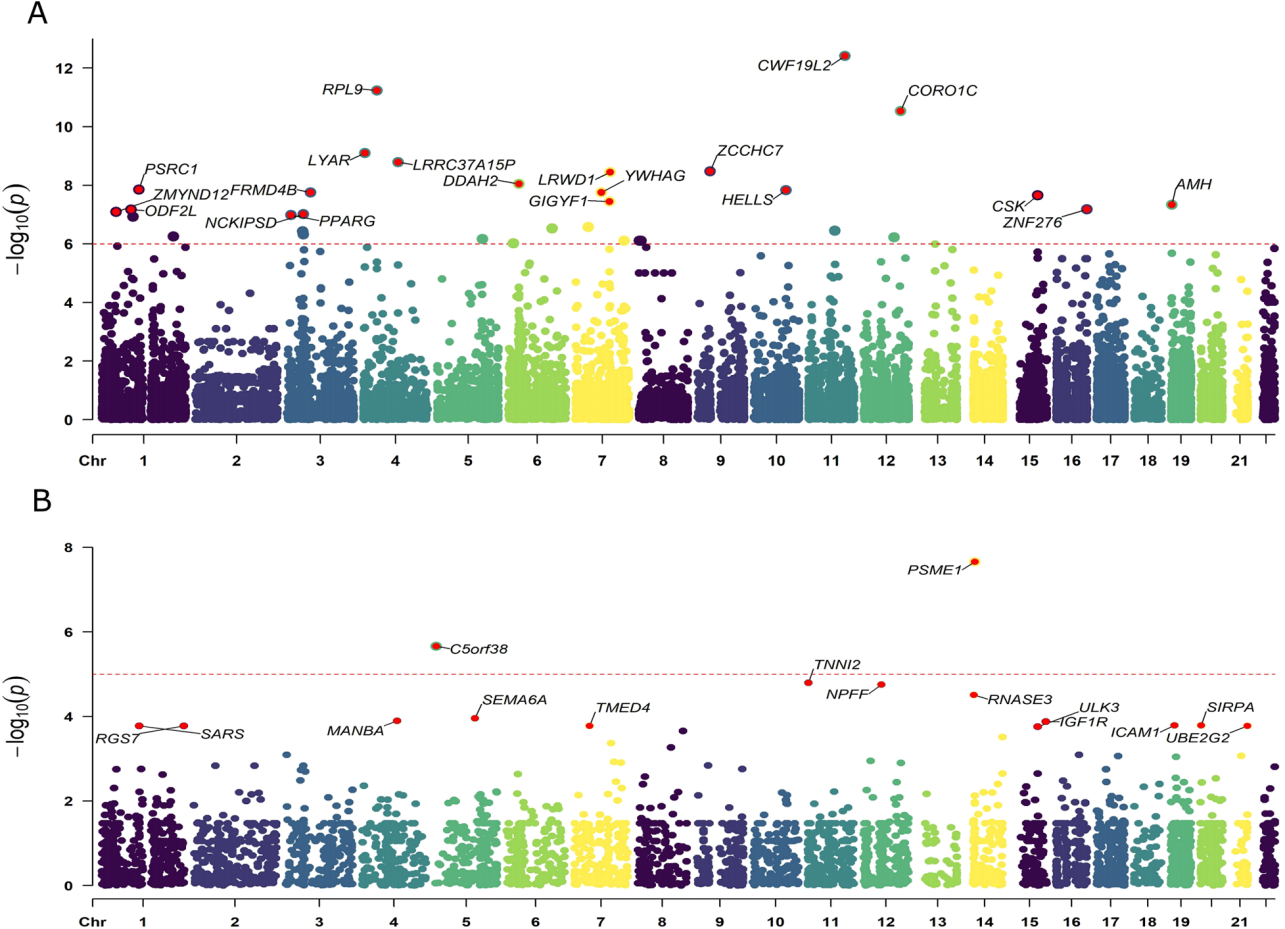
Manhattan plots illustrating the associations between exposure and CAC in MR analysis. **(A)** Associations between blood eQTLs and CAC. **(B)** Associations between pQTLs for circulating proteins with CAC. Labelled genes refer to MR results with FDR corrected *P* < 0.05; In panel **(A)**, only the 20 genes with the lowest statistical significance out of a total of 671 genes were labeled. Results were plotted at gene middle position.

**Figure 3:**
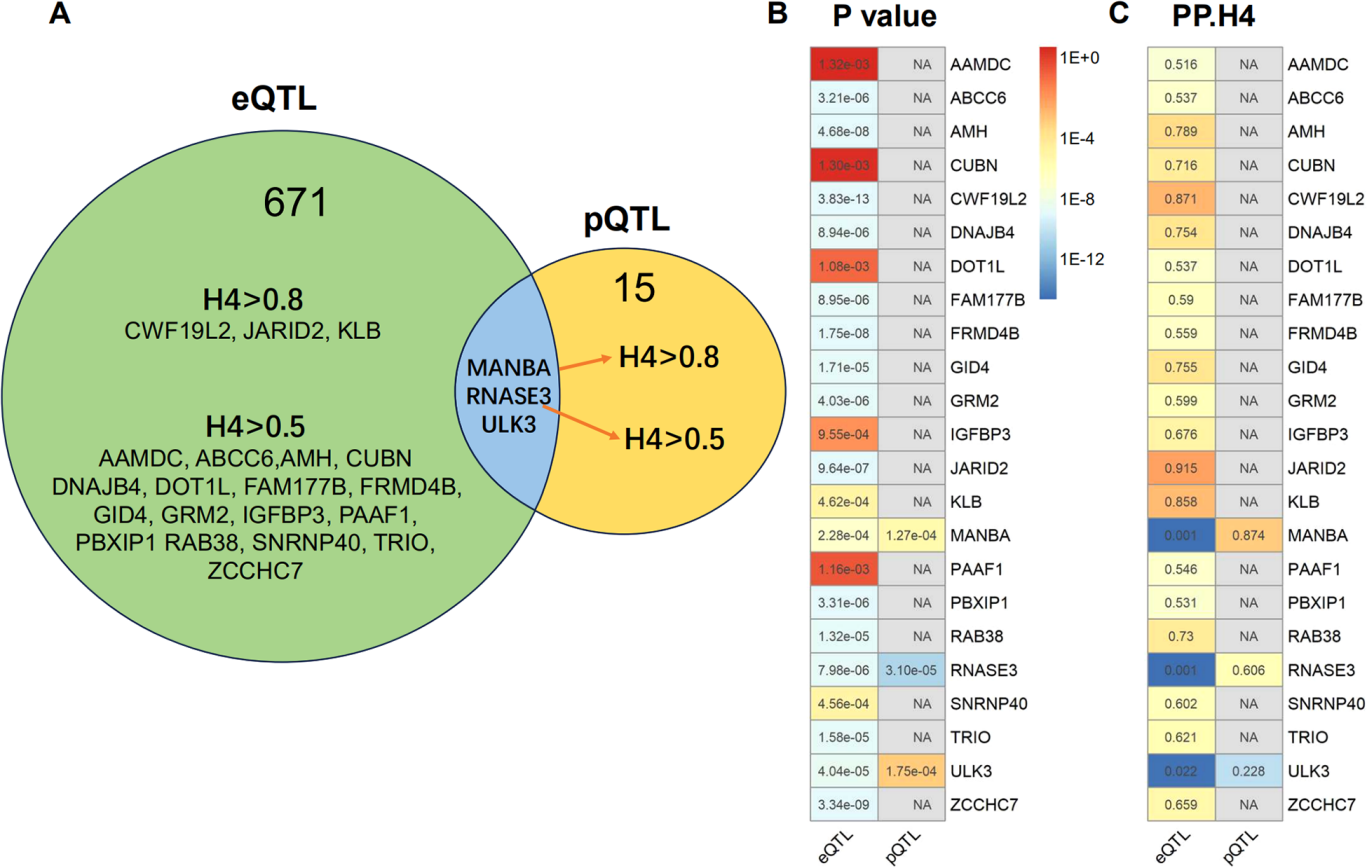
Summary of the results obtained from MR analysis and colocalization studies. **(A)** Venndiagram were constructed to visually represent the colocalization of both eQTL and pQTL MR results, with FDR-corrected threshold of *P* < 0.05. The term “H4” denotes the posterior probabilities of H4 (PP.H4) in this context. The presence of H4 > 0.5 indicates suggestive colocalization. The genes MANBA, RNASE3 and ULK3 exhibited FDR corrected *P* < 0.05 in both eQTL and pQTL MR analysis. The pQTL-based analysis revealed evidence of colocalization between MANBA and RNASE3, while no evidence of colocalization was observed for *ULK3*. **(B)** The MR *P*-values and heatmap of the 23 potential target gene. **(C)** The posterior probabilities of H4 for the 23 potential target genes.

We obtained eQTLs of 16,943 gene symbols from the eQTLGen dataset after clumping. Among these genes, 2,859 showed nominal association with CAC at a significance level of *P* < 0.05 (Figure 2A; Supplementary Table S4). A total of 671 genes were identified to be significantly associated with the risk of CAC based on FDR-adjusted *P*-value (<0.05). To validate the causal effect between eQTLs and CAC, we performed colocalization analysis which provided strong support for three genes (CWF19L2, JARID2, and KLB) showing high probability posterior probabilities (PP.H4 > 0.8). The expression levels of CWF19L2 and JARID2 were found to be positively associated with CAC [Beta 0.17, 95% confidence interval (CI) 0.12–0.22, *P* = 3.83E-13 for CWF19L2; Beta 0.2, 95% CI 0.12–0.27, *P* = 9.64E-07 for JARID2]. Conversely, KLB showed a negative association with CAC (Beta -1.4, 95% CI -2.19 to 0.62, *P* = 4.6E-04) (Table 1). Additionally, another 17 genes exhibited suggestive evidence of colocalization (PP.H4 > 0.5) (Figure 3; Supplementary Table S6).

**Table 1:** Summary of the results obtained from MR analysis and colocalization studies.

Exposure	Gene	Mendelian randomization			Colocalization	Categories of targets
		Beta	*P* value	FDR corrected *p* value	PP.H4	
eQTL	CWF19L2	0.17 (0.12–0.22)	3.83E-13	6.47E-09	0.871	Tier 2
	JARID2	0.2 (0.12–0.27)	9.64E-07	4.07E-04	0.915	Tier 2
	KLB	−1.4 (−2.19 to 0.62)	4.62E-04	0.020	0.858	Tier 2
	AAMDC	0.17 (0.07–0.27)	1.32E-03	0.039	0.516	Tier 3
	ABCC6	−0.42 (−0.6 to 0.24)	3.21E-06	8.3E-04	0.537	Tier 3
	AMH	0.34 (0.22–0.46)	4.68E-08	5.3E-05	0.789	Tier 3
	CUBN	−0.08 (−0.13 to 0.03)	0.001296	0.039	0.716	Tier 3
	DNAJB4	−0.3 (−0.43 to 0.17)	8.94E-06	1.5E-03	0.754	Tier 3
	DOT1L	−0.73 (−1.17 to 0.29)	1.08E-03	0.034	0.537	Tier 3
	FAM177B	0.22 (0.13–0.32)	8.95E-06	1.5E-03	0.59	Tier 3
	FRMD4B	0.18 (0.12–0.24)	1.75E-08	2.5E-05	0.559	Tier 3
	GID4	−0.29 (−0.42 to 0.16)	1.71E-05	2.2E-03	0.755	Tier 3
	GRM2	−0.24 (−0.34 to 0.14)	4.03E-06	9.9E-04	0.599	Tier 3
	IGFBP3	−0.13 (−0.21 to 0.05)	9.55E-04	0.032	0.676	Tier 3
	PAAF1	−0.07 (−0.12 to 0.03)	1.16E-03	0.036	0.546	Tier 3
	PBXIP1	−0.39 (−0.55 to 0.22)	3.31E-06	8.3E-04	0.531	Tier 3
	RAB38	0.09 (0.05–0.13)	1.32E-05	1.8E-03	0.730	Tier 3
	SNRNP40	−0.27 (−0.42 to 0.12)	4.56E-04	2.0E-02	0.602	Tier 3
	TRIO	0.53 (0.29–0.77)	1.58E-05	2.1E-03	0.621	Tier 3
	ZCCHC7	0.3 (0.2–0.39)	3.34E-09	8.7E-06	0.659	Tier 3
pQTL	MANBA	0.37 (0.18–0.56)	1.3E-04	0.049	0.874	Tier 2
	RNASE3	−0.24 (−0.35 to 0.13)	3.1E-05	0.026	0.606	Tier 3
Both	ULK3^a^	−0.14 (−0.20 to 0.07)	4.0E-05	0.004	0.022	Tier 3
	ULK3^b^	−0.67 (−1.02 to 0.32)	1.7E-04	0.049	0.228	

^a^ The Mendelian randomization and colocalization results were derived from the eQTL dataset.

^b^ The Mendelian randomization and colocalization results were derived from the pQTL dataset.

### pQTL based analysis

A total of 4,412 proteins with pQTL were included in the analysis. Among them, we identified 732 proteins that exhibited nominal significance (*P* < 0.05) (Figure 2B; Supplementary Table S5). Following multiple testing correction, a subset of 15 proteins demonstrated significant associations with the risk of CAC (FDR *P* < 0.05). The protein MANBA exhibited a positive association with CAC (Beta 0.37, 95% CI 0.18–0.56, *P* = 1.3E-04), and survived colocalization analysis (PP.H4 = 0.874). Conversely, RNASE3 showed a negative association with CAC (Beta -0.24, 95% CI -0.35 to 0.13, *P* = 3.1E-05), and there was suggestive evidence of colocalization (PP.H4 = 0.606) (Supplementary Table S7; Table 1).

### Summary of findings

The eQTL and pQTL based MR analysis revealed significant associations between three genes (MANBA, RNASE3, and ULK3) and CAC. Notably, the direction of association with CAC is consistent for these three genes (Table 1; Figure 3). The colocalization analysis provided support for the presence of both MANBA and RNASE3, while ULK3 did not exhibit any evidence of colocalization (PP.H4 = 0.022 using eQTL instruments, PP.H4 = 0.228 using pQTL instrument). A total of 23 genes were identified as potential therapeutic targets for CAC (Table 1).

### Replication analysis in abdominal aorta calcification dataset

**Figure 4:**
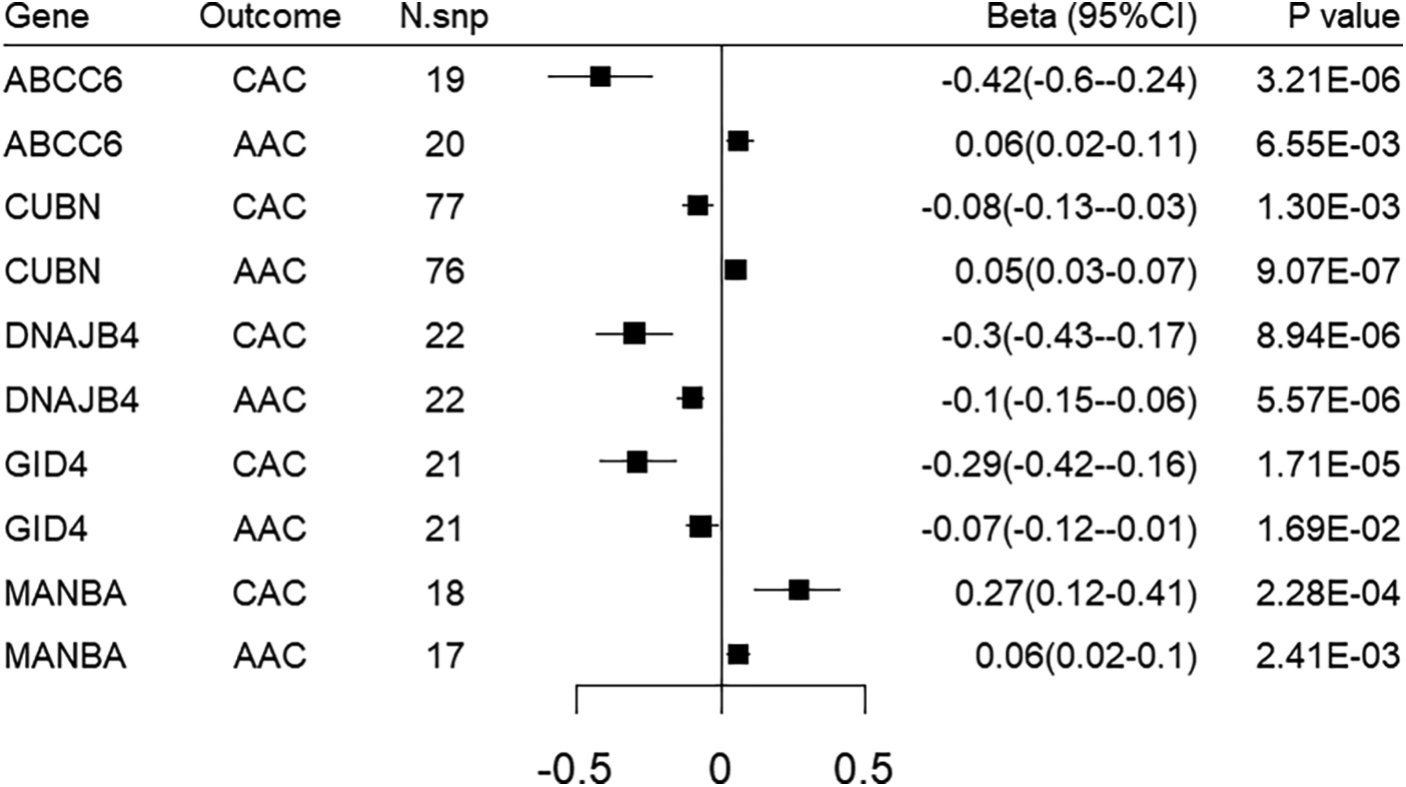
Forest plot illustrating the MR results of replication analysis in abdominal aorta calcification. CAC, coronary artery calcification; AAC, abdominal aorta calcification.

We sought to replicate all the identified target genes in an independent dataset of abdominal aorta calcification, namely the UK biobank imaging cohort. Genetically-predicted expression of 5 genes were replicated using either the Wald ratio or IVW method. Among them, DNAJB4 and CUBN demonstrated significant associations with FDR < 0.05, while GID4, MANBA, ABCC6 showed nominal significance. However, it is worth noting that the directionality of MR associations for ABCC6 and CUBN does not align with that observed for CAC MR associations (Figure 4; Supplementary Table S8).

### Phenome-wide association study

In the phenome-wide association study, ULK3, IGFBP3, DOT1L, and CWF19L2 were found to be associated with blood pressure. MANBA, FRMD4B, and CUBN were each associated with eGFR, pulse rate, and lipids, respectively. FAM177B was identified as being associated with coronary artery disease and myocardial infarction (Supplementary Table S9).

### Mouse knock-out models for potential target genes

We searched the IMPC and MGI resources for knock-out (KO) mice models to identify evidence of phenotype relevant to CAC resulting from target modification. Among the 23 genes examined, KO models of JARID2, KLB, DNAJB4, DOT1L, and IGFBP3 exhibited phenotypes in the cardiovascular system, such as abnormal heart development, abnormal placenta vasculature, increased heart weight, elevated cardiomyocyte apoptosis, decreased cardiac output, and thickened ventricular wall. Additionally, KO models of JARID2, TRIO, and ULK3 exhibited homeostasis and metabolism phenotypes, including increased circulating creatine, decreased circulating magnesium, impaired glucose tolerance, and increased circulating cholesterol. Furthermore, KO models of ABCC6 demonstrated phenotypes associated with calcinosis, calcified skin, and calcified artery, suggesting an intrinsic role in metastatic calcification (Supplementary Table S10).

### Druggability of identified therapeutic targets

We conducted a comprehensive search in the DGIdb Database to explore drug-gene interactions and identify potential therapeutic targets. From the dataset, we successfully identified seven out of the 23 genes as promising candidates for drug targeting (Table 2). Notably, none of these genes were found to be associated with CAC as drug targets. Interestingly, our analysis revealed several drugs that specifically target ABCC6, including one approved for osteoarthritis treatment. Drugs targeting AMH were primarily designed for sex hormone replacement therapy. Furthermore, we discovered that a specific drug targeting DOT1L functions as an antihypertensive agent. Three drugs designed to treat nonalcoholic fatty liver disease (NAFLD) were found to effectively target KLB. Two ULK3-targeting drugs were identified, one of which has gained approval for the treatment of chronic myeloid leukemia, while another has demonstrated potential in mitigating cardiac ischemia/reperfusion injury. Two drugs targeting IGFBP3 were approved for neoplasm treatment and selective COX-2 inhibition, respectively. Several drugs targeting GRM2, including antipsychotic agents, were identified. No drug-gene interaction information was available for GID4, DNAJB4, JARID2, FAM177B, FRMD4B, PAAF1, TRIO, SNRNP40, CWF19L2, PBXIP1, ZCCHC7, RAB38, CUBN, MANBA, RNASE3, and AAMDC.

**Table 2:** Druggability of potential target genes.

Target gene	Gene name	Drug name	Indications	Interaction type	Stage
ABCC6	ATP-binding cassette, sub-family C member 6	Docetaxel anhydrous, Thalidomide	Antineoplastic agent		Approved
		Heparan sulfate			
		Chondroitin sulfates	Osteoarthritis		Approved
AMH	Anti-Mullerian hormone	Testosterone	Hormone replacement		Approved
		Ethinyl estradiol	Hormone replacement		Approved
		Diethylstilbestrol	Contraceptive		Approved
		Tretinoin	Acne		Approved
DOT1L	DOT1 like histone lysine methyltransferase	Candesartan cilexetil	Antihypertensive agent		Approved
GRM2	Glutamate receptor, metabotropic 2	Decoglurant, LY404039, MGS-0210, Oleoyl-estrone, LY2979165, Opiate, LY-2300559			
		LY2140023	Antipsychotic agent		
		JNJ-40411813	Antipsychotic agent	Agonist	
		LY2979165, LY2969822, Pomaglumetad methionil		Agonist	
IGFBP3	Insulin-like growth factor binding protein 3	Fluorouracil	Antineoplastic agent		Approved
		Celecoxib	NSAID		Approved
KLB	Klotho beta	Pegbelfermin		Agonist	Clinical trial
		Aldafermin		Activator	Clinical trial
		Fazpilodemab		Agonist	
ULK3	Unc-51-like kinase 3	Hesperadin		Inhbitor	
		Imatinib	Antineoplastic agents		Approved

Overall, drugs targeting IGFBP3, ABCC6, ULK3, DOT1L, KLB, and AMH are promising candidates for drug repurposing. A previous genome-wide study identified IGFBP3 as a causal factor on CAC (14). ABCC6 and KLB have been found to be associated with calcification in previous literature. In this study, we identified ULK3, DOT1L, and AMH as novel targets for drug repurposing for CAC.

## Discussion

The integrated MR analysis of transcriptomic and proteomic data identified 23 potential target genes for CAC. Subsequent downstream analysis identified IGFBP3, ABCC6, ULK3, DOT1L, KLB, and AMH as promising candidates for drug repurposing.

IGFBP3, a member of the insulin-like growth factor (IGF) binding protein family, circulates in the plasma and interacts with IGFs to extend their half-life and modulate their interaction with cell-surface receptors. Previous studies have demonstrated an association between IGFBP3 and blood pressure traits (29). In a small clinical study, serum concentration of IGFBP3 was significantly correlated with coronary arteriosclerosis (30). A previous genome-wide study identified IGFBP3 as a causal factor on CAC (14). Consistent with these findings, our study provides compelling evidence of an inverse association between IGFBP3 and the risk of CAC. In addition, given that IGFBP3 is a target of antineoplastic and non-steroid anti-inflammatory drugs, our results suggest promising therapeutic opportunities for targeting IGFBP3 in the treatment of CAC.

The ABCC6 protein belongs to the superfamily of ATP-binding cassette transporters. Dysfunction of ABCC6 is the primary cause of pseudoxanthoma elasticum and generalized arterial calcification of infancy (GACI) (31). Extensive literature supports the pivotal role of ABCC6 in soft tissue calcification (16). ABCC6 facilitates the cellular efflux of ATP from liver and other tissues, which is rapidly converted by ENPP1 to pyrophosphate (PPi), a potent inhibitor of mineralization and ectopic calcification (32). Our study provides compelling evidence for a reverse association between ABCC6 and CAC. Drugs targeting the ABCC6/ENPP1/PPi axis may hold significant therapeutic potential for vascular calcification.

ULK3 is a serine/threonine protein kinase that functions as a pivotal regulator of Sonic hedgehog (SHH) signaling and autophagy. It is involved in keratinocyte self-renewal and tumorigenesis (33). However, limited information exists regarding the association between ULK3 and cardiovascular disease as well as CAC. In knockout mouse models, ULK3 was found to be associated with elevated levels of circulating creatine and cholesterol phenotypes in homeostasis and metabolism system, both of which are known risk factors for vascular calcification. Despite limited colocalization support, both eQTL and pQTL-based MR analysis revealed a reverse association between ULK3 and CAC. Therefore, further investigations are warranted to explore the relationship between ULK3 and calcification.

DOT1L is the sole H3K79 methyltransferase identified in human cells, playing pivotal roles in transcriptional regulation. Dysregulation of DOT1L has significant implications for mixed-lineage leukemia (34). Additionally, DOT1L is involved in modulating lipid biosynthesis and inflammatory responses in macrophages, thereby promoting stability of atherosclerotic plaque (35). Moreover, it modulates the gene expression of vascular smooth muscle cells and subsequently confers protection against the development of atherosclerosis (36). The process of osteogenic differentiation in vascular smooth muscle cells is crucial for arterial calcification (37). Based on our study findings, we observed an inverse association between DOT1L and the risk of CAC. This novel correlation between DOT1L and calcification warrants further investigation.

In the current study, a causal association between KLB and risk of CAC was observed. Three drugs targeting KLB were identified for potential treatment of NAFLD. However, downstream PheWAS and KO mouse model analysis did not provide evidence supporting an association with cardiovascular phenotypes. However, a recent meta-analysis evaluated the association between NAFLD and coronary artery disease. The study revealed a significant correlation between NAFLD and an increased risk of coronary artery disease (RR: 1.21, 95% CI: 1.07, 1.38), presence of CAC (CAC > 0, RR: 1.39, 95% CI: 1.15, 1.69), as well as the presence of calcified coronary plaques (RR: 1.55, 95% CI: 1.05, 2.27) (38). Preclinical studies also reveal that KLB can be a target of vascular calcification (17). Therefore, drugs targeting KLB may possess potential for repurposing.

AMH is primarily implicated in local ovarian follicle recruitment and development, while also exhibiting the ability to enhance osteoblast differentiation and calcification (39). The positive causal effect observed in this study further supports the potential pathophysiological role of AMH in vascular calcification. However, contrasting findings were reported in a cohort study involving 3,108 females, which demonstrated that a decline in circulating AMH levels may actually increase cardiovascular risk (40). Inconsistencies exist between AMH and cardiovascular risk factors and disease (41).

This study possesses several notable strengths. First, we employed MR and colocalization to identify potential targets for CAC. The utilization of MR design effectively minimized bias arising from confounding factors, while the application of colocalization enabled the detection of pleiotropic effect resulting from linkage disequilibrium. Second, we integrated the largest available dataset encompassing circulating transcriptomic and proteomic profiles as exposure variables in order to investigate the association between genetically proxied gene expression and CAC.

Some important limitations should be acknowledged. First, the estimates obtained from MR reflect the lifelong effects of genetic variants, which differ from those of drugs. Drugs are typically administered later in life and have a more pronounced short-term impact. Second, the study population were predominantly of European ancestry. Third, it is worth noting that colocalization analysis may lack sufficient power to detect potential causal effects, particularly when posterior probabilities (H4 and H3) are very low.

## Conclusion

The present study has successfully identified 23 causal genes associated with CAC. Through the integration of drug database and downstream analysis, IGFBP3, ABCC6, ULK3, DOT1L, KLB, and AMH have been prioritized as potential drug targets that warrant further investigation. Of these, ULK3, DOT1L, and AMH was recognized as novel targets for drug repurposing for CAC. These findings hold promise for future advancements in CAC drug development.

## Data Availability

The original contributions presented in the study are included in the article/Supplementary Material, further inquiries can be directed to the corresponding author.
